# Risk factors for tube exposure following Baerveldt glaucoma implantation and overlying tissue thickness assessed by anterior segment optical coherence tomography

**DOI:** 10.1007/s10384-026-01366-9

**Published:** 2026-04-22

**Authors:** Natsumi Murata, Sachi Kojima, Yuji Takihara, Eri Takahashi, Kei-Ichi Nakashima, Toshihiro Inoue

**Affiliations:** Department of Ophthalmology, Faculty of Life Sciences, Kumamoto University, 1-1-1 Honjo, Chuo-ku, Kumamoto, 860-8556 Japan

**Keywords:** Glaucoma drainage device, Baerveldt glaucoma implantation, Tube exposure, Anterior chamber, Anterior segment optical coherence tomography

## Abstract

**Purpose:**

We identified clinical factors associated with tube exposure following Baerveldt glaucoma implantation (BGI) and used anterior segment optical coherence tomography (AS-OCT) to assess postoperative tissue thickness over the tube.

**Study design:**

Retrospective, single-center cohort study.

**Methods:**

In this single-center retrospective cohort study, 405 eyes underwent BGI (BG101-350) with preserved scleral patch grafts between October 2014 and December 2022. Tube exposure was documented through clinical records and slit-lamp examination. Risk factors were evaluated using Firth’s penalized logistic regression. Overlying tissue thickness at 1 year postoperatively was measured via AS-OCT, and its association with clinical variables was analyzed using linear regression.

**Results:**

The mean follow-up was 630.7 days, and tube exposure occurred in 12 eyes (2.96%). Neovascular glaucoma (NVG) (odds ratio [OR] 5.03, p = 0.033) and anterior chamber (AC) tube insertion (OR 6.07, p = 0.017) were independently associated with exposure. Overlying tissue was significantly thinner with AC insertion compared with ciliary sulcus or pars plana placement (Kruskal–Wallis with Bonferroni-adjusted Dunn’s tests; AC vs sulcus, p = 0.039; AC vs pars plana, p < 0.001). Among eyes with AC insertion, prior micropulse transscleral cyclophotocoagulation (MP-CPC) was associated with greater tissue thickness (estimate 0.173; p = 0.032).

**Conclusion:**

AC insertion and NVG were associated with an increased risk of tube exposure after BGI. AS-OCT revealed thinner tissue over AC-placed tubes, providing a structural basis for this risk. Avoiding AC insertion may reduce exposure; the observed association between prior MP-CPC and thicker tissue requires prospective validation.

**Supplementary Information:**

The online version contains supplementary material available at https://doi.org/10.1007/s10384-026-01366-9.

## Introduction

Glaucoma drainage devices were first introduced with the Molteno implant and later expanded to include the Ahmed Glaucoma Valve, Baerveldt Glaucoma Implant [1], and PAUL Glaucoma Implant [2]. They are now indispensable in the management of refractory or complex glaucoma [3]. These devices function by diverting aqueous humor from the anterior chamber (AC) or posterior chamber through a tube to a subconjunctival plate. Tube exposure remains one of the most serious postoperative complications, with reported incidence rates of 0.9–15% [4–10]. This complication establishes a direct conduit for infection, substantially elevating the risk of endophthalmitis, which can lead to severe inflammation, hypotony, and irreversible vision loss [11, 12].

Standard practice is to cover the tube with a patch graft to reduce the risk of exposure, and several studies confirm its effectiveness [7]. However, biological graft materials progressively thin over time, and patch grafting does not provide complete protection [13–15]. Previous studies identify potential risk factors for tube exposure, including younger age, diabetes mellitus, neovascular glaucoma (NVG), ocular inflammation, and the absence of patch graft coverage [4–10, 12]. However, these findings have been inconsistent, likely due to variations in surgical techniques, implant models, and graft materials. In addition, the low incidence of exposure and the presence of clinical confounders complicate efforts to establish definitive causal relationships.

To address these uncertainties, we conducted a study following a standardized surgical protocol with Baerveldt BG101-350 implants and preserved scleral patch grafts. We investigated clinical factors associated with postoperative tube exposure. In addition, based on the hypothesis that progressive tissue thinning precedes exposure, we used anterior segment optical coherence tomography (AS-OCT) to assess tissue thickness at 1 year postoperatively and evaluated its association with clinical variables.

## Methods

### Study design and participants

This retrospective study included patients who underwent Baerveldt glaucoma implantation (BGI) at Kumamoto University Hospital between October 2014 and December 2022. All surgery used the BG101-350 model with preserved scleral patch grafts. Tube exposure was identified from clinical records and confirmed by slit-lamp examination. The study was conducted in accordance with the principles of the Declaration of Helsinki and approved by the Institutional Review Board of Kumamoto University Hospital. This study was retrospective, and written informed consent (IC) was waived. Instead, we used an opt-out approach via our institution’s website. This approach disclosed study information to patients and ensured their right to decline participation.

### Surgical procedure

All procedures were performed by multiple surgeons using the BG101-350 device (Abbott Medical Optics). Under sub-Tenon’s anesthesia, a fornix-based conjunctival flap was created in either the superior or inferior temporal quadrant. To prevent early hypotony, the tube was ligated with 8-0 polyglactin, and the plate was positioned beneath the rectus muscles and secured to the sclera 8–10 mm posterior to the limbus. The tube insertion site—AC, ciliary sulcus, or pars plana—was selected according to the ocular condition. A 23-gauge needle was used to create the entry tract, through which the tube was inserted and anchored to the sclera with an 8-0 nylon suture. Sherwood slits were created using an 8-0 nylon needle to permit gradual aqueous outflow before ligature dissolution.

A preserved donor scleral patch graft was placed over the tube, and the conjunctiva was closed with 8-0 polyglactin sutures. At the conclusion of surgery, a subconjunctival betamethasone injection was administered. Postoperative medications included 1.5% levofloxacin for 1 month and 0.1% betamethasone for 3 months.

### Tube exposure analysis

Tube exposure was defined as direct visualization of the tube through the conjunctiva on slit-lamp examination. Clinical data were retrospectively extracted, and the following variables were analyzed: age, sex, laterality, systemic hypertension, diabetes mellitus, previous ocular procedures (cataract surgery, trabeculotomy, trabeculectomy, vitrectomy, selective laser trabeculoplasty, and micropulse transscleral cyclophotocoagulation [MP-CPC]), combined procedures, glaucoma type (primary open-angle glaucoma, exfoliation glaucoma [EXG], uveitic glaucoma, NVG, and others), tube insertion site, preoperative intraocular pressure, number of medications, and implantation quadrant. MP-CPC was performed using a CYCLO G6^™^ micropulse P3 probe (IRIDEX) according to the manufacturer’s instructions [16]. MP-CPC was conducted in patients who required further intervention for elevated IOP or visual field progression and for whom incisional surgery was not a viable option. The other glaucoma category comprised childhood (n = 17), angle-closure (n = 9), steroid-induced (n = 7), traumatic (n = 7), pigmentary (n = 1), familial amyloid polyneuropathy-associated (n = 5), cataract-related (n = 3) (elevated IOP after cataract surgery, elevated IOP after intrascleral fixation of an IOL, and aphakic eye after congenital cataract surgery), and oil-induced (n = 2) cases. Univariate associations were evaluated using Fisher’s exact test and the Wilcoxon rank-sum test. Variables deemed clinically relevant were assessed in a multivariable logistic regression model.

### Tissue thickness analysis

**Fig. 1:**
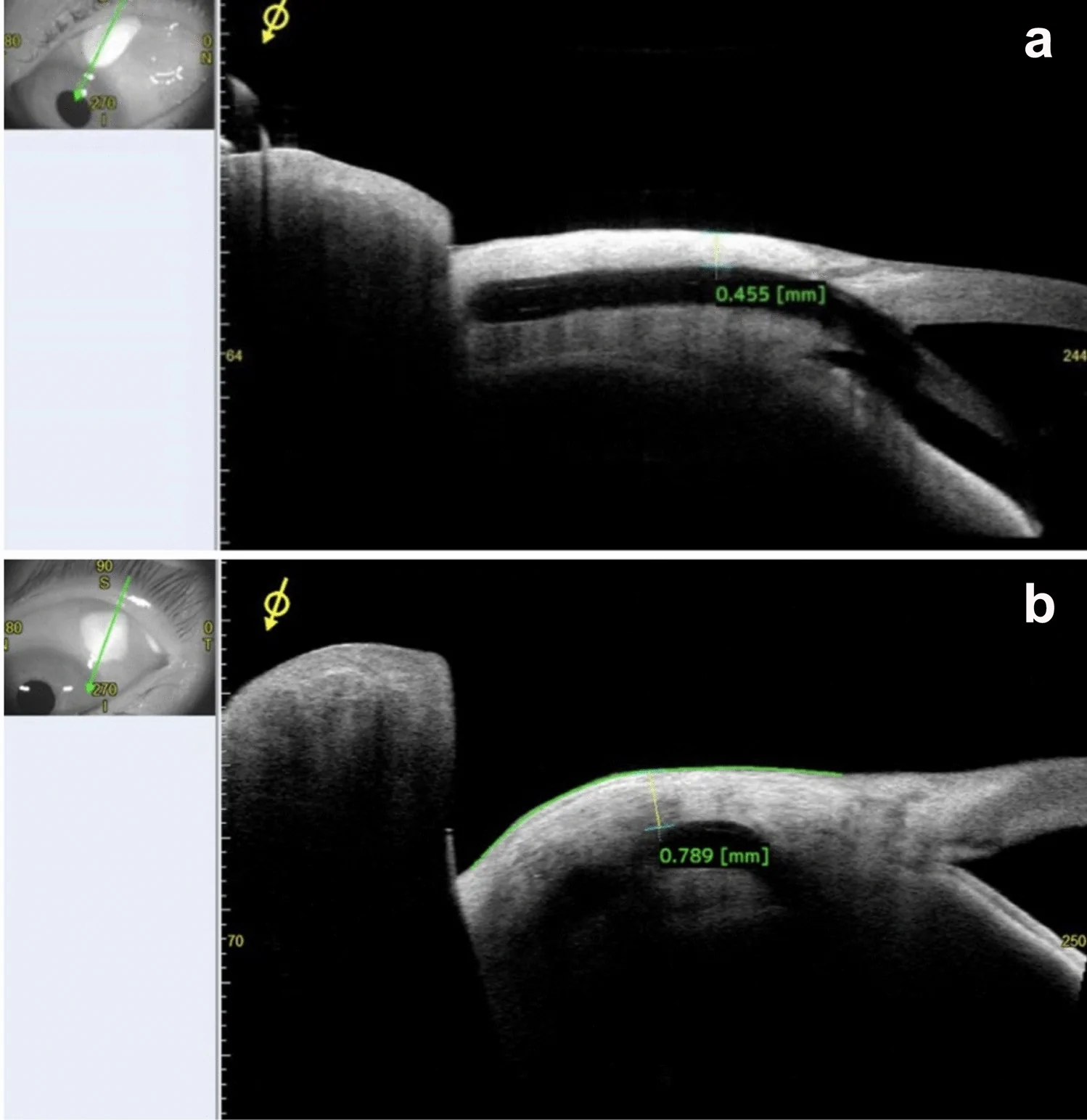
Cross-sectional AS-OCT images obtained using the CASIA2 system. **a** The tube is inserted into the AC, and **b** the tube is inserted through the pars plana into the vitreous cavity. Tissue thickness was defined as the vertical distance from the conjunctival surface to the outer edge of the tube at the scleral entry site. Green calliper lines indicate the measurement. *AC* anterior chamber; *AS-OCT* anterior segment optical coherence tomography

Patients with analyzable AS-OCT images obtained using CASIA2 (Tomey Corp.) at approximately 1 year (± 3 months) postoperatively were included. Images were analyzed in 2D mode with cross-sections oriented perpendicular to the tube. Because distinct boundaries between conjunctiva, Tenon’s capsule, and graft material were not consistently discernible, tissue thickness at the tube entry site was defined as the distance from the conjunctival surface to the outer edge of the tube, measured with the built-in caliper (Fig. 1). All measurements were performed by a single examiner (N.M.). Subsequently, clinically relevant variables were analyzed using a multivariable linear regression model.

### Statistical analysis

Statistical analyses were performed using R (version 4.4.2; R Foundation for Statistical Computing). Univariate associations were evaluated using Fisher’s exact test and the Wilcoxon rank-sum test. Variables deemed clinically relevant were assessed in a multivariable logistic regression model. Group comparisons were conducted using the Wilcoxon rank-sum test for continuous variables and Fisher’s exact test for categorical variables. Considering the rarity of tube exposure, Firth’s penalized logistic regression was applied to model risk factors. Associations with tissue thickness were evaluated using linear regression and nonparametric tests, including the Kruskal–Wallis test with Bonferroni-adjusted Dunn’s post hoc analysis and the Wilcoxon rank-sum test. P values were interpreted in conjunction with clinical relevance rather than as strict thresholds for inclusion in multivariable models.

## Results

### Study population

**Fig. 2:**
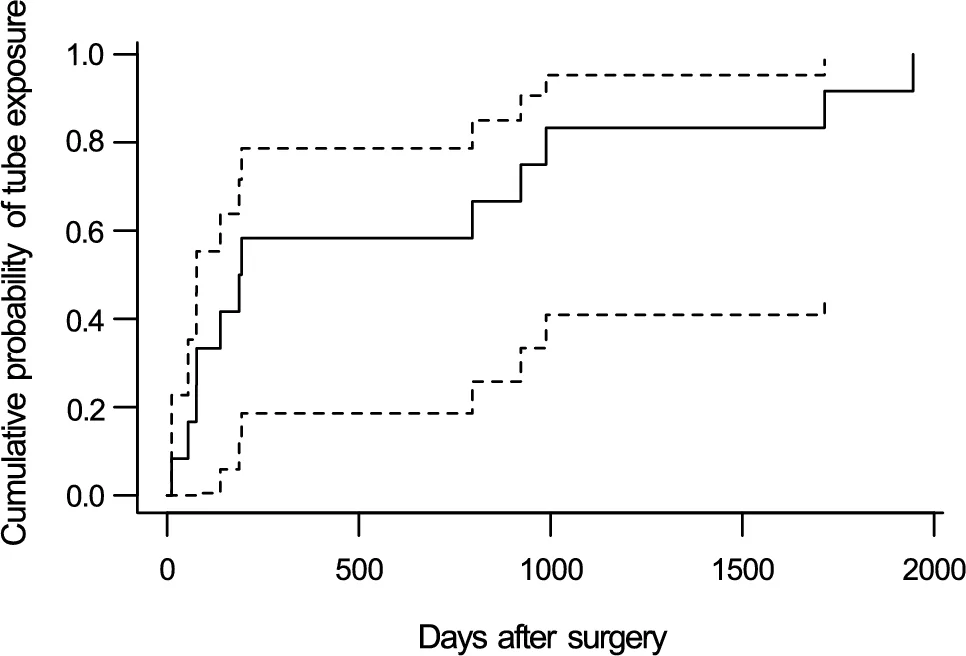
Kaplan–Meier curve for time to tube exposure following Baerveldt glaucoma implantation (n = 12). The solid line indicates the estimated cumulative proportion of exposure events, and the dashed lines represent 95% confidence intervals

In total, 405 eyes from 358 patients underwent BGI with the BG101-350 model and preserved scleral patch grafts. Table 1 presents the demographics and clinical characteristics of the study participants. Data are presented as mean ± standard deviation (SD), median, or number (%), as appropriate. The mean follow-up duration was 630.7 ± 493.1 (median, 426) days. Tube exposure occurred in 12 eyes (2.9%); the interval from surgery to exposure ranged from 12 to 1945 (mean, 592; median, 191) days. Figure 2 shows the Kaplan–Meier curve for time to tube exposure, where the solid line represents the cumulative probability of exposure and dashed lines indicate 95% confidence intervals (CIs). More than half of the exposure events occurred within the first postoperative year. The clinical characteristics of the 12 eyes with postoperative tube exposure are detailed in Table S1 of the Online Resource.

**Table 1:** Baseline characteristics and univariate comparison of clinical parameters between eyes with and without tube exposure

	All (n=405)	Exposure group (n=12)	Non-exposure group (n=393)	*p*-value^1^
Observation days, mean ± SD	630.70 ± 493.08	592.16 ± 679.56	631.89 ± 487.38	0.252
Age (years), mean ± SD	67.48 ± 16.93	70.17 ± 12.18	67.40 ± 17.05	0.858
Male, n (%)	255 (63%)	7 (58.3%)	248 (63.1%)	0.767
Systemic disease, n (%)				
HT	188 (46.4%)	5 (41.7%)	183 (46.6%)	0.778
DM	95 (23.5%)	4 (33.3%)	91 (23.2%)	0.487
Preoperative status, mean ± SD				
IOP (mmHg)	29.89 ± 8.69	28.50 ± 4.98	29.93 ± 8.78	0.802
Number of medications	4.30 ± 0.90	4.25 ± 0.96	4.31 ± 0.90	0.872
Previous surgeries				
Cataract surgery, n (%)	253 (62.5%)	7 (58.3%)	246 (62.6%)	0.769
Trabeculotomy, n (%)	113 (27.9%)	4 (33.3%)	109 (27.7%)	0.745
Trabeculectomy, n (%)	173 (42.7%)	5 (41.7%)	168 (42.7%)	1.000
Vitrectomy, n (%)	42 (10.4%)	0 (0.0%)	42 (10.7%)	0.622
Previous SLT, n (%)	22 (5.4%)	1 (8.3%)	21 (5.3%)	0.493
Previous MP-CPC, n (%)	14 (3.4%)	0 (0.0%)	14 (3.6%)	1.000
Concomitant surgeries, n (%)				
Cataract surgery	61 (15.1%)	2 (16.7%)	59 (15%)	0.699
Vitrectomy	7 (1.7%)	0 (0.0%)	7 (1.8%)	1.000
Glaucoma diagnosis, n (%)				
POAG	93 (23.0%)	1 (8.3%)	92 (23.4%)	0.311
EXG	139 (34.3%)	5 (41.7%)	134 (34.1%)	0.555
UG	77 (19.0%)	3 (25.0%)	74 (18.8%)	0.707
NVG	45 (11.1%)	3 (25.0%)	42 (10.7%)	0.138
Others	51 (12.6%)	0 (0.0%)	51 (13%)	0.377
Tube insertion sites, n (%)				
Anterior chamber	263 (64.9%)	11 (91.7%)	252 (64.1%)	0.064
Sulcus	98 (24.2%)	1 (8.3%)	97 (24.7%)	0.308
Pars plana	44 (10.9%)	0 (0.0%)	44 (11.2%)	0.378
Implant position (superior temporal), n (%)	345 (85.2%)	9 (75.0%)	336 (85.5%)	0.398
Laterality (right), n (%)	213 (52.6%)	6 (50.0%)	207 (52.7%)	1.000

“Others” includes childhood (n = 17), angle-closure (n = 9), steroid-induced (n = 7), traumatic (n = 7), pigmentary (n = 1), FAP-associated (n = 5), cataract-related (n = 3), and oil-induced (n = 2) glaucoma

*DM* diabetes mellitus; *EXG* exfoliation glaucoma; *FAP* familial amyloid polyneuropathy; *HT* hypertension; *IOP* intraocular pressure; *MP-CPC* micropulse transscleral cyclophotocoagulation; *NVG* neovascular glaucoma; *POAG* primary open-angle glaucoma; *SD* standard deviation; sulcus, ciliary sulcus; *SLT* selective laser trabeculoplasty; *UG* uveitic glaucoma

^1^The Wilcoxon rank-sum test was used for quantitative variables, and Fisher’s exact test for qualitative variables

### Tube exposure analysis

Table 1 presents the clinical characteristics of eyes with (n = 12) and without (n = 393) tube exposure. Continuous variables were compared using the Wilcoxon rank-sum test, and categorical variables were compared using Fisher’s exact test. Univariate analyses were exploratory and not corrected for multiple testing; therefore, statistically significant findings should be interpreted cautiously and confirmed in multivariable models. No variables reached statistical significance (p < 0.05), although NVG and AC tube insertion demonstrated relatively low p-values and were included in a multivariable logistic regression model.

**Fig. 3:**
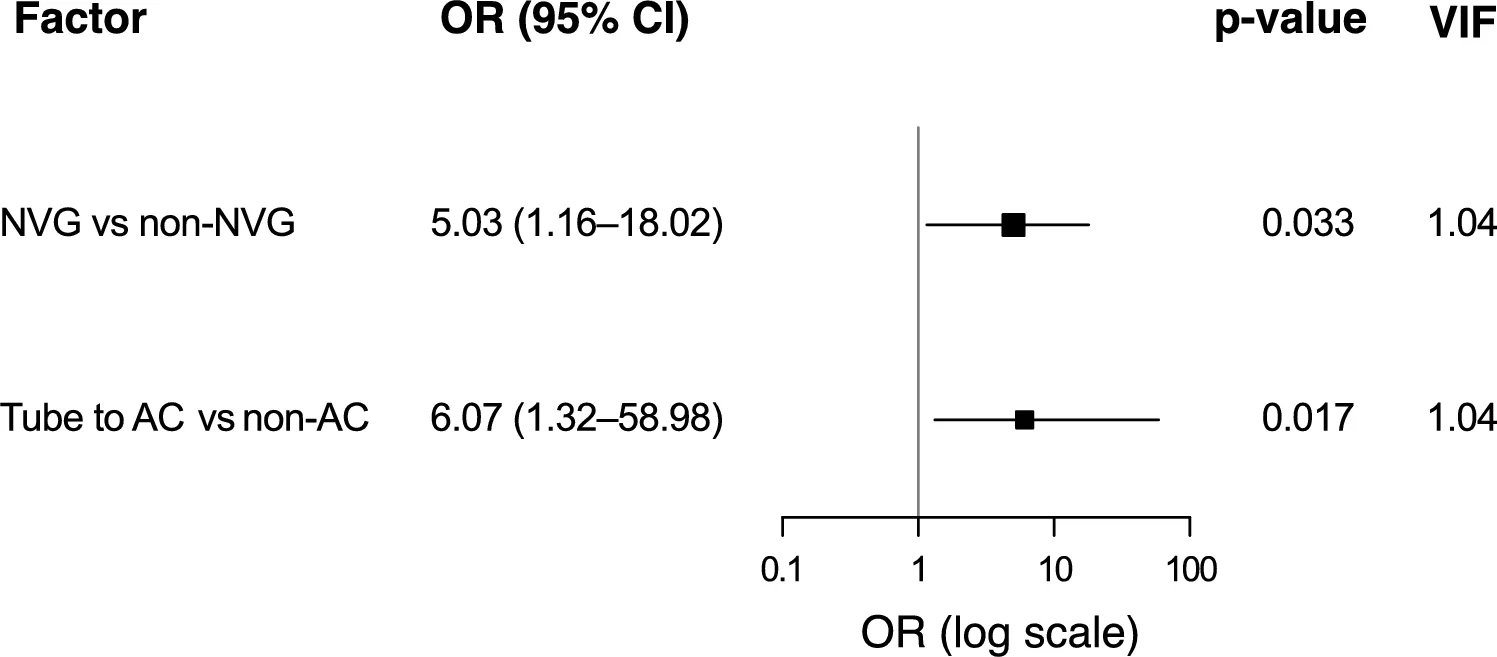
Forest plot of multivariable logistic regression using Firth’s penalized likelihood. NVG and AC tube insertion were independently associated with tube exposure risk. ORs are plotted on a logarithmic scale with 95% confidence intervals. The right-most column lists each variable’s VIF, all < 2, indicating no substantial multicollinearity. *AC* anterior chamber; *NVG* neovascular glaucoma; *OR* odds ratio; sulcus, ciliary sulcus; *VIF* variance inflation factor

To reduce the risk of overfitting given the small number of exposure events, the multivariable model was limited to two clinically plausible variables and analyzed using Firth’s penalized likelihood logistic regression, which is appropriate for rare-event data (Fig. 3). Both NVG (odds ratio [OR] 5.03; 95% CI 1.16–18.02; p = 0.033) and AC tube insertion (OR 6.07; 95% CI 1.32–58.98; p = 0.017) were independently associated with increased exposure risk. Variance inflation factors for all predictors were < 2, indicating no problematic multicollinearity. Despite the use of penalized regression, wide CIs reflect residual uncertainty. Subgroup univariate analysis of eyes with AC tube insertion implied a potential association between NVG and exposure (p = 0.027), which was not statistically significant in multivariable analysis (OR 5.55; p = 0.078) (Table S2 and Fig. S1 of the Online Resource).

### Tissue thickness analysis

**Fig. 4:**
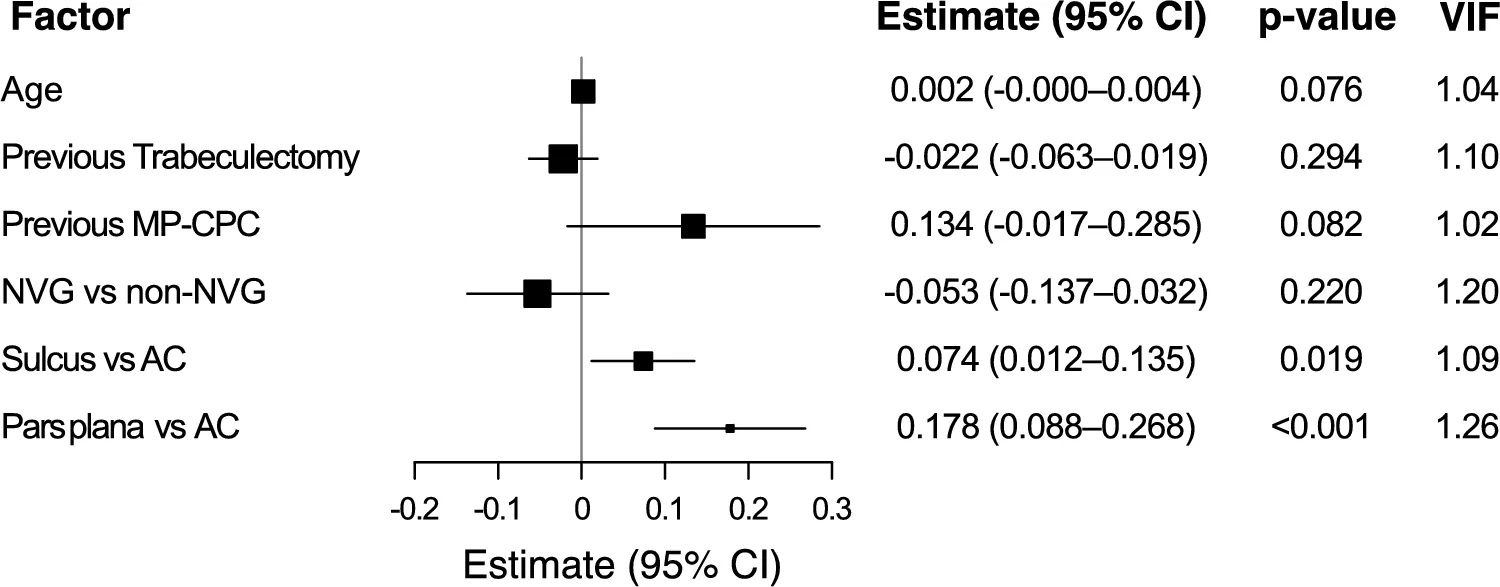
Forest plot of multivariable linear regression for factors associated with tissue thickness over the tube at 1 year postoperatively. Ciliary sulcus (sulcus) and pars plana insertions were each associated with significantly greater thickness than AC insertion. Estimates and 95% confidence intervals are plotted on a linear (not logarithmic) scale. The right-most column lists each predictor’s VIF, all < 2, indicating that multicollinearity was not a concern. *AC* anterior chamber; *VIF* variance inflation factor

Table 2 presents the demographic and clinical characteristics of the eyes included in the tissue thickness analysis. AS-OCT was performed at approximately 1 year postoperatively in 200 eyes from 178 patients. Comparisons of baseline characteristics showed no meaningful differences between this cohort and the overall tube exposure cohort. Considering the exploratory nature of this analysis, univariate tests were not corrected for multiple comparisons. Univariate linear regression identified associations between tissue thickness and age (p = 0.016), previous trabeculectomy (p = 0.032), MP-CPC (p = 0.135), and tube insertion site (sulcus: p = 0.009; pars plana: p < 0.001) compared with AC insertion group (reference) (Table 2). These variables, along with NVG, were entered into a multivariable linear regression model (Fig. 4). Compared with AC insertion, sulcus tube insertion (estimate, 0.071; p = 0.023) and pars plana insertion (estimate, 0.176; p < 0.001) were independently associated with greater tissue thickness. Variance inflation factors for all predictors were < 2, indicating no problematic multicollinearity.

**Table 2:** Baseline characteristics of the AS-OCT thickness analysis population and univariate analysis of factors associated with tissue thickness over the tube

	Summary	Estimate^1^	95% CI	*p*-value^2^
Age (years), mean ± SD	68.88 ± 13.99	0.002	0.000–0.004	0.016
Male, n (%)	125 (62.5%)	– 0.014	– 0.07–0.042	0.615
Systemic disease, n (%)				
HT	91 (45.5%)	0.006	– 0.048–0.060	0.824
DM	51 (25.5%)	0.047	– 0.015–0.109	0.133
Preoperative status, mean ± SD				
IOP (mmHg)	29.81 ± 8.97	0.001	– 0.002–0.004	0.344
Number of medications	4.49 ± 0.74	– 0.010	– 0.047–0.026	0.572
Previous surgeries				
Cataract surgery, n (%)	125 (62.5%)	0.051	– 0.004–0.107	0.069
Trabeculotomy, n (%)	54 (27.0%)	– 0.002	– 0.063–0.059	0.955
Trabeculectomy, mean ± SD	72 (36.0%)	– 0.061	– 0.117–0.005	0.032
Vitrectomy, n (%)	22 (11.0%)	0.131	0.047–0.216	0.003
Previous SLT, n (%)	16 (8.0%)	– 0.006	– 0.106–0.094	0.907
Previous MP-CPC, n (%)	6 (3.0%)	0.120	– 0.038–0.278	0.135
Concomitant surgeries, n (%)				
Cataract surgery	29 (14.5%)	– 0.008	– 0.085–0.068	0.828
Vitrectomy	2 (1.0%)	– 0.098	– 0.37–0.173	0.475
Glaucoma diagnosis, n (%)				
POAG	52 (26.0%)	– 0.017	– 0.078–0.045	0.598
EXG	67 (33.5%)	0.009	– 0.048–0.067	0.747
UG	39 (19.5%)	– 0.018	– 0.086–0.050	0.604
NVG	25 (12.5%)	0.011	– 0.071–0.092	0.799
Others	17 (8.5%)	0.035	– 0.061–0.132	0.472
Tube insertion sites, n (%)				
Anterior chamber	126 (63.0%)	(Reference)		
Sulcus	51 (25.5%)	0.082	0.021–0.143	0.009
Pars plana	23 (11.5%)	0.164	0.081–0.247	<0.001
Implant position (superior temporal), n (%)	173 (86.5%)	– 0.019	– 0.098–0.060	0.633
Laterality (right), n (%)	107 (53.5%)	0.027	– 0.027–0.081	0.333

“Others” includes childhood (n = 3), angle-closure (n = 4), steroid-induced (n = 3), traumatic (n = 3), pigmentary (n = 1), FAP-associated (n = 1), cataract-related (n = 1), and oil-induced (n = 1) glaucoma

*DM* diabetes mellitus; *EXG* exfoliation glaucoma; *FAP* familial amyloid polyneuropathy; *HT* hypertension; *IOP* intraocular pressure; *MP-CPC* micropulse transscleral cyclophotocoagulation; *NVG* neovascular glaucoma; *POAG* primary open-angle glaucoma; *SD* standard deviation; sulcus, ciliary sulcus; *SLT* selective laser trabeculoplasty; *UG* uveitic glaucoma

^1^Estimates

^2^p-values were derived from simple linear regression

**Fig. 5:**
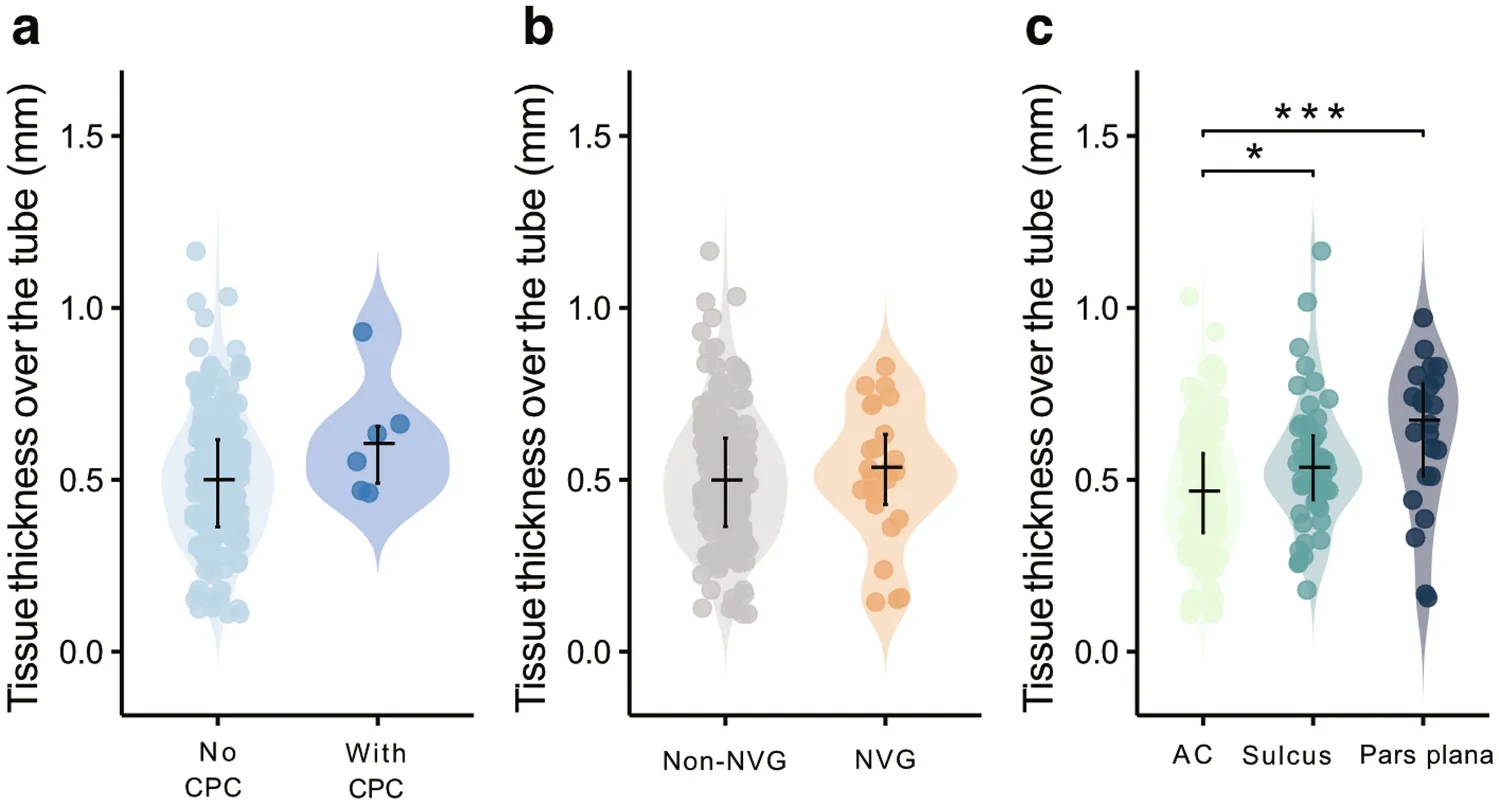
Violin plots of tissue thickness over the tube at 1 year postoperatively. Violin plots showing tissue-thickness distributions at 1 year postoperatively (± 3 months), stratified by: **a** previous MP-CPC status (No MP-CPC, n = 194; With MP-CPC, n = 6), **b** NVG status (Non-NVG, n = 175; NVG, n = 25), and **c** tube insertion site (AC, n = 126; sulcus, n = 51; pars plana, n = 23). Statistical comparisons were performed using the Wilcoxon rank-sum test for two-group analyses or the Kruskal–Wallis test with Bonferroni-adjusted Dunn’s post hoc test for three-group analyses. Data are presented as medians (IQR). Dunn’s pairwise comparisons (C): AC vs sulcus, adjusted p = 0.039; AC vs pars plana, adjusted p < 0.001; sulcus vs pars plana, adjusted p = 0.218 (not significant). *adjusted p < 0.05; **adjusted p < 0.01; ***adjusted p < 0.001. *AS-OCT* anterior segment optical coherence tomography; *AC* anterior chamber; *IQR* interquartile range; *MP-CPC* micropulse transscleral cyclophotocoagulation; NVG, neovascular glaucoma; sulcus, ciliary sulcus

Kruskal–Wallis analysis followed by Bonferroni-adjusted Dunn’s post hoc testing demonstrated significant differences in tissue thickness across insertion sites (p = 0.046). Pairwise comparisons revealed significantly thinner tissue in the AC group compared with both the sulcus (adjusted p = 0.039) and pars plana groups (adjusted p < 0.001) (Fig. 5).

**Fig. 6:**
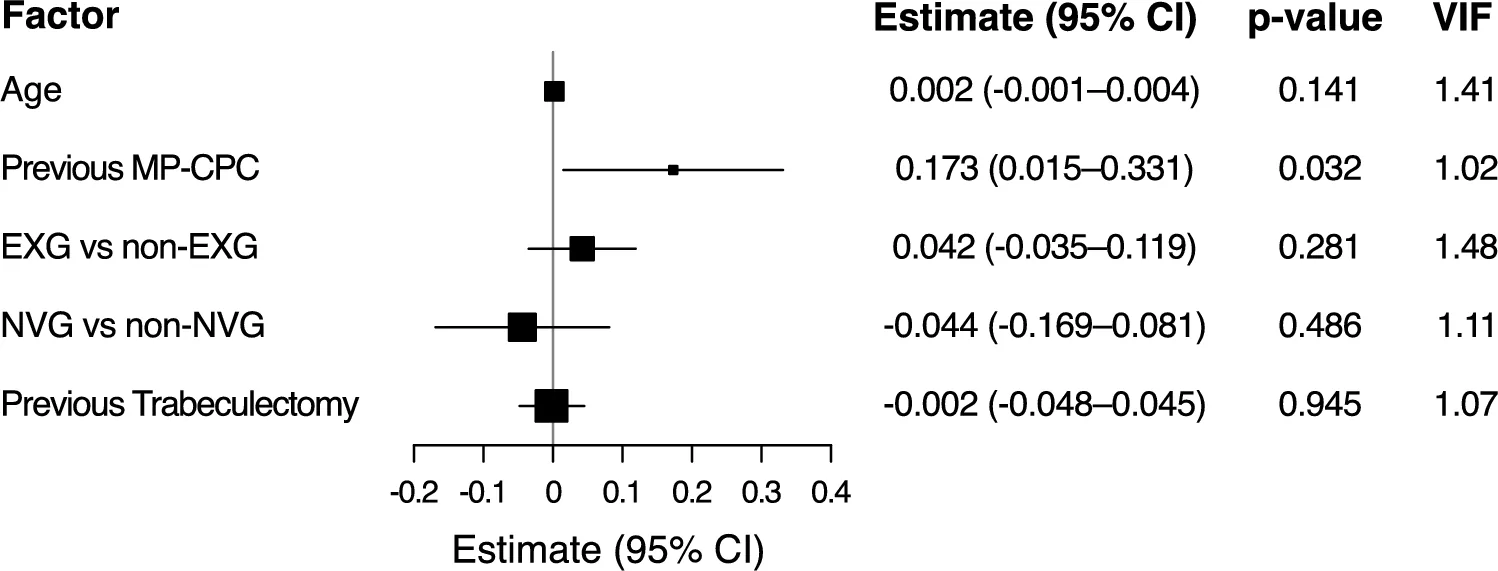
Forest plot of multivariable linear regression in eyes with AC insertion. Previous MP-CPC was associated with greater tissue thickness (estimate: 0.173; 95% CI 0.015–0.331; p = 0.032), while other factors were not significant. Estimates and 95% confidence intervals are plotted on a linear scale. The right-most column shows each predictor’s VIF, all < 2, indicating no problematic multicollinearity. *AC* anterior chamber; *MP-CPC* micropulse transscleral cyclophotocoagulation; *CI* confidence interval; *VIF* variance inflation factor

In a subgroup analysis of eyes with AC tube insertion, univariate linear regression implied associations between tissue thickness and age (p = 0.010), previous MP-CPC (p = 0.017), and EXG (p = 0.019) (Table S3 of the Online Resource). Multivariable linear regression indicated that previous MP-CPC was independently associated with greater tissue thickness (estimate, 0.173; p = 0.032) (Fig. 6). The interval between MP-CPC and BGI was 381.2 ± 270.1 days (mean ± standard deviation), ranging from 56 to 1072 days. Violin plots illustrate the distribution of tissue thickness by MP-CPC status, with eyes having prior MP-CPC demonstrating significantly thicker tissue compared with those without (p = 0.027, Wilcoxon rank-sum test; Fig. S2 of the Online Resource).

## Discussion

In this study, AC tube insertion was identified as an independent risk factor for tube exposure following BGI using a standardized protocol with preserved scleral patch grafts. Although prior studies have included tube insertion site as a descriptive variable, few have directly examined its association with exposure risk [8, 17]. The use of a standardized surgical protocol in our cohort allowed for a more precise evaluation of this factor.

Mechanical microtrauma from blinking, particularly near the limbus, contributes to conjunctival thinning and subsequent tube exposure [18]. AC insertion positions the tube closer to the limbus, increasing its exposure to mechanical stress. Progressive thinning of biological patch grafts is well documented in glaucoma drainage device surgery [11, 13, 15], and OCT-based studies demonstrate that thinner grafts are associated with higher exposure risk. For instance, Schipper et al. report that eyes developing exposure had thinner pericardial grafts at 3 and 6 months postoperatively compared to controls [15]. Notably, exposure may sometimes be avoided even after complete graft resorption if conjunctival integrity is maintained. These findings support a two-step model in which graft thinning is followed by conjunctival erosion. In our study, AC insertion appeared to accelerate both graft thinning and conjunctival erosion, likely due to the increased mechanical stress associated with limbal proximity.

NVG was associated with tube exposure, consistent with prior studies [6, 10]; however, it was not correlated with graft thickness in our study. This finding implies that NVG may impair conjunctival healing rather than directly accelerate graft degradation. Similarly, systemic ischemic conditions, such as ischemic heart disease, have been associated with an increased risk of glaucoma drainage device exposure, likely due to compromised tissue repair [14]. In contrast, we observed no significant association between exposure and other factors, including younger age, female sex, inferior device placement, or prior ocular surgery [4–7, 10, 19]. The uniform use of preserved scleral grafts in our cohort mitigated the impact of these variables by buffering mechanical stress.

Graft thinning may be driven by mechanical stress and biological factors, such as enzymatic degradation and chronic inflammation, which can occur even in regions subjected to minimal mechanical friction [17]. In our subgroup analysis of eyes with AC tube insertion, previous MP-CPC was associated with significantly greater tissue thickness over the tube. MP-CPC is a transscleral laser procedure used to lower intraocular pressure in refractory glaucoma and may promote tissue preservation by minimizing postoperative inflammation, as it delivers energy in short bursts, reducing collateral thermal damage compared to continuous-wave cyclophotocoagulation [20–22]. Histological studies indicate that MP-CPC can still induce moderate subconjunctival fibrosis [23, 24], which may stabilize tissue via modulation of matrix metalloproteinases and their inhibitors, favoring reduced degradation [25–28]. Although our findings imply an association between prior MP-CPC and increased tissue thickness, the small number of cases and potential selection bias limit any inference of causality. Consequently, no definitive conclusions regarding a protective effect of MP-CPC can be drawn.

Even with standardized grafting, the tube insertion site remains a modifiable risk factor. Avoiding AC placement may reduce the incidence of exposure, particularly in high-risk eyes. Several studies suggest that pars plana placement or repositioning may lower the risk of exposure and recurrence [18, 29]. A case-control study by Alobaida et al. reports that ciliary sulcus insertion was associated with fewer severe complications—including exposure, corneal decompensation, endophthalmitis, and vision loss—compared to AC insertion [30]. Ciliary sulcus insertion has been associated with reduced endothelial cell loss without compromising efficacy [31–33]. Pars plana insertion maximizes the distance from the cornea but carries higher risks of posterior segment complications [34, 35]. Therefore, although sulcus or pars plana tube placement may offer advantages for reducing postoperative exposure, careful case selection is essential.

Moreover, Tenon’s capsule advancement during trabeculectomy is reported to improve filtration bleb formation [36]. Tenon’s capsule advancement was not performed in our BGI procedures. Tenon’s capsule advancement during BGI may reduce the risk of postoperative exposure.

Our study has several limitations. First, the number of tube exposure events was small (n = 12), which may have reduced the precision of our multivariable estimates and increased the risk of overfitting. Although we applied Firth’s penalized logistic regression to address this issue, the wide confidence intervals indicate residual uncertainty. Second, the retrospective design may have introduced selection bias and unmeasured confounding. Third, tissue thickness was assessed at a single 1-year time point, precluding evaluation of longitudinal tissue remodeling. Fourth, potential confounders, such as selective pars plana insertion or the use of MP-CPC in high-risk cases, were not captured. Finally, as a single-center study conducted in a Japanese population, the findings may not be generalizable to other ethnicities or surgical settings. Considering the low incidence of postoperative tube exposure, conducting a sufficiently powered prospective study would be logistically and ethically challenging. Despite these limitations, our study provides valuable insights that may generate hypotheses and inform future research and clinical decision-making.

In conclusion, AC tube insertion and NVG were independently associated with an increased risk of postoperative tube exposure, even in eyes receiving preserved scleral patch grafts. AS-OCT imaging demonstrated that AC placement was linked to significantly thinner overlying tissue, providing a structural explanation for the heightened risk. These findings emphasize the importance of individualized surgical planning and imply that alternative insertion sites, such as the ciliary sulcus or pars plana, may be preferable in high-risk eyes. Although prospective validation is warranted, our study offers clinically actionable insights for preventing postoperative tube exposure.

## Supplementary Information

Below is the link to the electronic supplementary material.

Supplementary file1 (DOCX 208 KB)
