# Supplementary material for: Risk factors for tube exposure following Baerveldt glaucoma implantation and overlying tissue thickness assessed by anterior segment optical coherence tomography
Source: Jpn J Ophthalmol. 2026 Apr 22;70(5):1208–17. doi: 10.1007/s10384-026-01366-9 (PMC13616009; doi:10.1007/s10384-026-01366-9)
Supplement: Supplementary file 1 [file 10384_2026_1366_MOESM1_ESM.docx]

**Table S1. Clinical characteristics of eyes with tube exposure following Baerveldt glaucoma implantation**

| **Age** | **Diagnosis** | **Tube insertion site** | **Implant position** | **Days to Exposure** | **HbA1c (Surgery)** | **HbA1c (Exposure)** |
| --- | --- | --- | --- | --- | --- | --- |
| 64 | EXG | AC | inferior | 1945 |  |  |
| 70 | UG | AC | superior | 988 |  |  |
| 52 | NVG (inflammatory) | AC | superior | 796 | 6.5 | 5.9 |
| 47 | POAG | AC | superior | 1714 |  |  |
| 71 | NVG (CRVO) | AC | superior | 922 | 7.3 | 7.5 |
| 69 | UG | AC | superior | 76 |  |  |
| 68 | EXG | AC | superior | 12 |  |  |
| 77 | UG | AC | inferior | 139 |  |  |
| 71 | NVG (DR) | AC | superior | 194 | 6.2 | 6.9 |
| 85 | EXG | AC | superior | 188 |  |  |
| 87 | EXG | AC | superior | 55 |  |  |
| 83 | EXG | CS | inferior | 77 |  |  |

Data are presented for the 12 eyes that developed tube exposure. For cases with neovascular glaucoma (NVG), HbA1c levels at the time of surgery and around the time of exposure are provided.

Abbreviations*: AC, anterior chamber; CRVO, central retinal vein occlusion; CS, ciliary sulcus; DR, diabetic retinopathy; EXG, exfoliation glaucoma; NVG, neovascular glaucoma; POAG, primary open angle glaucoma; UG, uveitic glaucoma.*

| **Table S2. Univariate comparison of clinical parameters in eyes with AC tube insertion.** | | | | |
| --- | --- | --- | --- | --- |
|  | | Exposure group (n=11) | Non-exposure group (n=252) | p-value* |
| Observation days, mean ± SD | | 639.00 ± 692.12 | 659.36 ± 492.68 | 0.378 |
| Age (years), mean ± SD | | 69.01 ± 12.06 | 67.11 ± 16.72 | 0.956 |
| Male/Female, n (%) | | 7/4 (63.6/36.4%) | 164/88 (65.1/34.9%) | 1.000 |
| Systemic disease, n (%) | |  |  |  |
|  | HT | 5 (45.5%) | 116 (46.0%) | 1.000 |
|  | DM | 4 (36.4%) | 48 (19.0%) | 0.236 |
| Preoperative status, mean ± SD | |  |  |  |
|  | IOP (mmHg) | 28.81 ± 5.09 | 29.87 ± 8.76 | 0.940 |
|  | Number of medications | 4.18 ± 0.98 | 4.28 ± 0.87 | 0.761 |
| Previous surgeries | |  |  |  |
|  | Cataract surgery, n (%) | 7 (63.6%) | 133 (52.8%) | 0.550 |
|  | Trabeculotomy, n (%) | 4 (36.4%) | 67 (26.6%) | 0.495 |
|  | Trabeculectomy, n (%) | 4 (36.4%) | 130 (51.6%) | 0.370 |
|  | Vitrectomy, n (%) | 0 (0.0%) | 3 (1.2%) | 1.000 |
| Previous SLT, n (%) | | 1 (9.1%) | 13 (5.2%) | 0.459 |
| Previous MP-CPC, n (%) | | 0 (0.0%) | 10 (4.0%) | 1.000 |
| Concomitant surgeries, n (%) | |  |  |  |
|  | Cataract surgery | 2 (18.2%) | 37 (14.7%) | 0.670 |
|  | Vitrectomy | 0 (0.0%) | 1 (0.4%) | 1.000 |
| Glaucoma diagnosis, n (%) | |  |  |  |
|  | POAG | 1 (9.1%) | 69 (27.4%) | 0.298 |
|  | EXG | 4 (36.4%) | 96 (38.1%) | 1.000 |
|  | UG | 3 (27.3%) | 44 (17.5%) | 0.420 |
|  | NVG | 3 (27.3%) | 14 (5.6%) | 0.027 |
|  | Others | 0 (0.0%) | 29 (11.5%) | 0.616 |
| Implant position (superior temporal), n (%) | | 9 (81.8%) | 206 (81.7%) | 1.000 |
| Laterality (right), n (%) | | 6 (54.5%) | 132 (52.4%) | 1.000 |
| Abbreviations: AC, anterior chamber; DM, diabetes mellitus; EXG, exfoliation glaucoma; FAP, familial amyloid polyneuropathy; HT, hypertension; IOP, intraocular pressure; MP-CPC, micropulse transscleral cyclophotocoagulation; NVG, neovascular glaucoma; POAG, primary open-angle glaucoma; SD, standard deviation; sulcus, ciliary sulcus; SLT, selective laser trabeculoplasty; UG, uveitic glaucoma | | | | |
| *The Wilcoxon rank-sum test was used for quantitative variables, and Fisher’s exact test for qualitative variables. | | | | |

| **Table S3. Univariate analysis of the clinical factors associated with tissue thickness in the AC-insertion subgroup.** | | | | | |
| --- | --- | --- | --- | --- | --- |
|  | | Summary | Estimate* | Lower 95% CI | p-value* |
| Age (years), mean ± SD | | 67.59 ± 14.75 | 0.003 | 0.001–0.005 | 0.010 |
| Male/Female, n (%) | | 77 (61.1%) | -0.011 | -0.076–0.054 | 0.744 |
| Systemic disease, n (%) | |  |  |  |  |
|  | HT | 55 (43.7%) | 0.009 | -0.055–0.073 | 0.773 |
|  | DM | 25 (19.8%) | 0.005 | -0.074–0.085 | 0.898 |
| Preoperative status, mean ± SD | |  |  |  |  |
|  | IOP (mmHg) | 29.52 ± 8.84 | 0.001 | -0.003–0.004 | 0.699 |
|  | Number of medications | 4.48 ± 0.76 | -0.026 | -0.068–0.016 | 0.218 |
| Previous surgeries | |  |  |  |  |
|  | Cataract surgery, n (%) | 65 (51.6%) | 0.016 | -0.048–0.079 | 0.622 |
|  | Trabeculotomy, n (%) | 31 (24.6%) | 0.015 | -0.058–0.089 | 0.679 |
|  | Trabeculectomy, mean ± SD | 0.56 ± 0.69 | -0.014 | -0.060–0.033 | 0.564 |
|  | Vitrectomy, n (%) | 0 (0.0%) | NA | NA | NA |
| Previous SLT, n (%) | | 10 (7.9%) | -0.018 | -0.135–0.099 | 0.764 |
| Previous MP-CPC, n (%) | | 5 (4.0%) | 0.194 | 0.035–0.352 | 0.017 |
| Concomitant surgeries, n (%) | |  |  |  |  |
|  | Cataract surgery | 20 (15.9%) | 0.015 | -0.071–0.102 | 0.725 |
|  | Vitrectomy | 1 (0.8%) | -0.095 | -0.452–0.262 | 0.600 |
| Glaucoma diagnosis, n (%) | |  |  |  |  |
|  | POAG | 35 (27.8%) | -0.041 | -0.111–0.030 | 0.256 |
|  | EXG | 46 (36.5%) | 0.077 | 0.013–0.142 | 0.019 |
|  | UG | 27 (21.4%) | -0.014 | -0.091–0.064 | 0.728 |
|  | NVG | 9 (7.1%) | -0.064 | -0.187–0.058 | 0.300 |
|  | Others | 9 (7.1%) | -0.048 | -0.170–0.075 | 0.444 |
| Implant position (superior temporal), n (%) | | 105 (83.3%) | 0.010 | -0.095–0.075 | 0.816 |
| Laterality (right), n (%) | | 68 (54.0%) | 0.030 | -0.034–0.093 | 0.353 |
| Abbreviations: AC, anterior chamber; DM, diabetes mellitus; EXG, exfoliation glaucoma; FAP, familial amyloid polyneuropathy; HT, hypertension; IOP, intraocular pressure; MP-CPC, micropulse transscleral cyclophotocoagulation; NVG, neovascular glaucoma; POAG, primary open-angle glaucoma; SD, standard deviation; sulcus, ciliary sulcus; SLT, selective laser trabeculoplasty; UG, uveitic glaucoma. | | | | | |
| *Estimates and p-values were derived from simple linear regression. | | | | | |

**Fig. S1.** Forest plot of multivariable logistic regression in the AC-insertion subgroup. NVG was not statistically significant (p = 0.078) but showed a high OR. ORs are plotted on a logarithmic scale with 95% CIs. The right-most column lists each predictor’s VIF, all < 2, indicating no problematic multicollinearity.
Abbreviations: AC, anterior chamber; CI, confidence interval; NVG, neovascular glaucoma; OR, odds ratio; VIF, variance inflation factor.

**Fig. S2.** Violin plots showing tissue thickness in eyes with AC insertion, stratified by: (a) Previous MP-CPC status (No MP-CPC, n = 121; With MP-CPC, n = 5; p = 0.027), (b) EXG status (Non-EXG, n = 80; EXG, n = 46; p = 0.032), and (c) NVG status (Non-NVG, n = 117; NVG, n = 9). Statistical comparisons were performed using the Wilcoxon rank-sum test. Data are presented as median (IQR). *p < 0.05.
Abbreviations: AC, anterior chamber; CI, confidence interval; EXG, exfoliation glaucoma; NVG, neovascular glaucoma; MP-CPC, micropulse transscleral cyclophotocoagulation; IQR, interquartile range.
